# Functional Characterization of the Putative Hepatitis B Virus Core Protein Late Domain Using Retrovirus Chimeras

**DOI:** 10.1371/journal.pone.0072845

**Published:** 2013-08-29

**Authors:** Mayra L. Garcia, Tracy D. Reynolds, Walther Mothes, Michael D. Robek

**Affiliations:** 1 Department of Pathology, Yale University School of Medicine, New Haven, Connecticut, United States of America; 2 Department of Microbial Pathogenesis, Yale University School of Medicine, New Haven, Connecticut, United States of America; Drexel University College of Medicine, United States of America

## Abstract

The hepatitis B virus (HBV) Core protein encodes a late (L)-domain like motif (_129_PPAYRPPNAP^138^) that has been purported to serve as a docking site for recruitment of host factors such as Nedd4 that can mediate viral particle release from infected cells. However, mutation of this region of Core typically disrupts nucleocapsid formation in the cytoplasm, making it difficult to ascertain if the Core PPAY motif constitutes a functional L-domain that mediates HBV release in the context of replicating virus. Since many viral L-domains are functionally interchangeable between different virus families, and such swapping experiments have been used as a tool to identify other viral sequences with L-domain activity, we generated chimeric constructs between murine leukemia virus (MLV) Gag and HBV Core to determine if the potential HBV L-domain motif is sufficient to stimulate virus release. We found that the HBV Core PPAY motif, but not the PNAP motif, demonstrates L-domain activity in the context of MLV replication to direct virus release and infectious virion production. Additionally, we found that overexpression of the cellular Nedd4 or WWP1 ubiquitin ligases stimulates release of a partially defective PPAY domain mutant, providing further evidence supporting a role for the Nedd4 ubiquitin ligase in promoting HBV release. These studies lend further insight into the mechanisms used by HBV to mediate its release from infected cells.

## Introduction

Current therapies for chronic hepatitis B virus (HBV) infection include antiviral drugs that block HBV genome replication, such as adefovir and entecavir, or interferon-α (IFN-α), which stimulates the immune response for clearance of the virus [1], [2]. However, these treatments are limited by harmful side effects, inadequate availability in developing countries, costliness, and viral resistance. In order to identify new targets that are amenable to therapeutic development for this serious disease, the cellular mechanisms that govern HBV replication must be more completely understood. One aspect of the HBV life cycle that warrants further investigation is the method of HBV release from the infected cell. Emerging evidence indicates that the cellular ubiquitin (Ub) pathway plays a role in this process and therefore may be a potential therapeutic target.

Many viral structural proteins encode highly conserved late domain (L-domain) sequence motifs that mediate virus release [3]. While first described for the Retroviridae, this list has expanded to include members of other virus families such as the Rhabdoviridae [4], Filoviridae [5], [6], Arenaviridae [7], and Orthopoxviridae [8]. Detailed mutational analysis of viral L-domains have identified three classes of motifs that are essential for mediating viral release: P(T/S)AP, PPxY, and YxxL. L-domain activity within the viral structural protein can be attributed to motif-mediated protein-protein interactions with host proteins that facilitate access to the endocytic pathway for viral release. To mediate release of P(T/S)AP motif-containing viruses such as HIV-1 [9]–[12], Ebola [6], [11], and human T-cell leukemia virus type 1 (HTLV-1) [13], the motif interacts with the Src homology 3 (SH3) domain modules of the tumor suppressor gene (Tsg101) protein, a key ESCRT-I component for the recognition and sorting of ubiquitinated proteins to multivesicular body (MVB) internal vesicles [14]. In the case of Mason-Pfizer monkey virus (M-PMV) [15], HTLV-1 [13], [16], Ebola [5], [6], [17], VSV [18], MLV [19], and RSV [20], the PPxY motif is recognized by the WW domain-containing proteins of the Nedd4 family of Ub ligases [21]. The YxxL motif found in the structural proteins of EIAV [22]–[24], Sendai virus [25], and HIV-1 [23], [24], [26], is responsible for binding Alix (ALG-2-interacting protein X), an adapter protein that recruits the viral protein to the MVB by interacting with both Tsg101 of ESCRT-I and CHMP4 of ESCRT-III [23], [27]. To ensure access to the endocytic machinery, several viruses including HTLV-1 [13], [16], Ebola [28], HIV [29] and MLV [30] contain more than one type of motif within their structural proteins that display complimentary roles in mediating release.

The HBV Core protein contains two L-domain-like sequences located within a single motif: _129_ PPAYRPPNAP ^138^. The Nedd4 Ub ligase interacts with the Core L-domain-like PPAY motif and mutation of the tyrosine residue to alanine (Y132A) abolishes the Nedd4 interaction [31]. This evidence suggests that the Core L-domain-like motif, in a manner similar to that observed by other L-domain containing viruses, may serve as a docking site for recruitment of several host factors that mediate viral release. However, although the Core Y132A mutation disrupts the Nedd4/Core interaction, it is unclear if this motif constitutes a genuine L-domain. The PPAY motif is located in a region of Core that directs interdimer contacts during capsid formation [32], [33], and the Y132A mutation has previously proven to interfere with capsid formation [34]. Since mutations in this Core domain result in a replication-defective virus that does not assemble infectious virions, it is difficult to ascertain the role of the PPAY motif as an L-domain in the context of replicating HBV.

The observation that some viral L-domains are functionally interchangeable between heterologous viruses has led to the identification of new viral L-domain sequences and motif binding partners [35], [36]. In the case of some PPxY motif containing viruses, such experiments have been useful at identifying additional HECT ligases that bind the L-domain motif. Although Nedd4 is the prototypic member of the Nedd4 family of nine HECT Ub ligases [21], some viruses can utilize several different HECT ligases from the Nedd4 family, suggesting that they play functionally redundant roles in allowing viruses to gain access to the endocytic machinery [3]. For instance, the MLV PPPY L-domain motif can recruit WWP1, WWP2, Itch and Nedd4 to direct viral release [19], [30]. In addition to the L-domains themselves, the HECT domains of the Ub ligases are also functionally interchangeable, demonstrating another layer of flexibility in which the cellular endosomal sorting machinery can be utilized by viruses to mediate their release [37], [38].

Since viral L-domains are functionally interchangeable between different virus families, and complementation can be used as a tool to identify sequences with L-domain activity, we generated chimeric constructs between MLV Gag and HBV Core to determine if the two potential PPAY and PNAP L-domain motifs are sufficient to stimulate viral release as well as recruit cellular ubiquitin-dependent proteins of the endocytic pathway. We found that the Core PPAY motif, but not the PNAP motif, demonstrates L-domain activity in the context of MLV replication to direct infectious virion production. Furthermore, we provide additional evidence that the Nedd4 Ub ligase can promote virus release in an HBV L-domain dependent manner.

## Materials and Methods

### DNA Constructs and Mutagenesis

MLV GagPol plasmid, MLV GagPol4A plasmid, MLV Gag-YFP plasmid, Friend MLV Env plasmid, and MLV long terminal repeat-lacZ reporter construct (MLV LTR-LacZ) were previously described in Sherer et al. [39]. For generation of MLV Gag^Core^-YFP L-domain chimeras, pUC57 expression plasmids were synthesized (Genscript) to encode the MLV Gag gene with the endogenous L-domain sequence (nucleotides 382 to 393) replaced by the HBV Core L-domain sequence motif PPAY/PNAP (WIRTPPAYRPPNAPILST), PPAY (WIRTPPAYRP), or PNAP (RPPNAPILST). An *Xho*I fragment containing the MLV Gag^Core^ chimera gene sequence was subcloned into MLV Gag-YFP to generate MLV Gag^PPAY/PNAP^-YFP, MLV Gag^PPAY^-YFP, or MLV Gag^PNAP^-YFP expression constructs and a *Afl*III/*Xho*I DNA fragment containing the Core L-domain was subsequently subcloned into MLV GagPol to generate MLV GagPol^PPAY/PNAP^, MLV GagPol^PPAY^, and MLV GagPol^PNAP^ expression constructs, respectively. The MLV Gag^PPAY/PNAP^-YFP construct was used as a template for mutagenesis of the Core PPAY motif and an *Afl*III/*Xho*I DNA fragment was subsequently subcloned in MLV GagPol to generate MLV GagPol^LPAY/PNAP^, MLV GagPol^PPAF/PNAP^, or MLV GagPol^PPAA/PNAP^. HECT Ub ligase expression constructs used in this study that contained the full length (Nedd4, Nedd4L, WWP1, WWP2, Smurf1, Smurf2, Itch) or the truncated (Neddd4, Nedd4L, WWP1, Smurf2, Itch) versions of the proteins with YFP fused to the N-terminus were a gift from Paul Bieniasz (The Rockefeller University) and were previously described in Martin-Serrano et al. [19].

All mutations in the expression plasmids were introduced using the QuikChange II XL site-directed mutagenesis kit (Stratagene, La Jolla, CA) with the following primers and verified by sequencing: for the LPAY/PNAP mutation (MLV GagPol^LPAY/PNAP^), 5-GGA TTC GCA CTC TTC CAG CTT ATA GAC CAC CAA ATG-3; for the PPAF/PNAP mutation (MLV GagPol^PPAF/PNAP^), 5-GAT TCG CAC TCC TCC AGC TTT TAG ACC ACC AAA TG-3; for the PPAA/PNAP mutation (MLV GagPol^PPAA/PNAP^), 5-GAT TCG CAC TCC TCC AGC TGC TAG ACC ACC AAA TG-3.

### Cell Culture and Transfections

The avian DFJ8 cell line that stably expresses the murine ecotropic receptor MCAT-1 [40] and the human embryonic kidney 293T cell line [41] were cultured at 37°C in a humidified 5% CO_2_/air atmosphere in DMEM high glucose medium supplemented with 10% fetal bovine serum, 5 mM L-glutamine, 50 U of penicillin/ml, and 50 µg of streptomycin/ml. DFJ8 cells were supplemented with 200 µg/ml of G418. Transfections of 293T cells with plasmid DNA constructs were performed with Lipofectamine 2000 (Invitrogen) as recommended by the manufacturer.

### MLV Release Assay

293T cells were seeded in a 6-well plate (500,000 cells/well) and transfected the following day with wild type, chimeric, or mutant MLV GagPol plasmids, a Friend MLV Env expression plasmid, and a MLV LTR-LacZ reporter plasmid. In experiments where the ability of HECT Ub ligases or Ub ligase fragments were used to enhance or inhibit viral release, various amounts of YFP-fusion protein expression plasmid was cotransfected with the viral constructs as indicated. At 24 h after transfection, the cell culture medium was clarified by low speed centrifugation and subsequently filtered through a 0.45-µm-pore-size syringe filter before isolation of virus particles by centrifugation at 13,000 rpm for 60 min. After centrifugation, the supernatant was removed and the virus pellet was suspended in 50 µl NP-40 lysis buffer (1% [vol/vol] NP-40, 50 mM Tris [pH 7.5], 150 mM NaCl, and 1X protease inhibitor cocktail [Roche]) then boiled in 1X SDS sample buffer for 10 min at 95°C. Cells were washed with 1X PBS and lysed in 500 µl of NP-40 lysis buffer for 20 min on ice. Cell lysates were cleared by centrifugation at 13,000 rpm for 10 min, and the supernatant was collected and boiled in 1X SDS sample buffer for 10 min at 95°C. Proteins in cell or virus lysates were separated on a 10% SDS acrylamide gel and transferred to a nitrocellulose membrane. The membranes were probed with a rat monoclonal anti-MLV CA antibody (1∶1000) or a rabbit polyclonal anti-GFP antibody (1∶4000; Abcam), followed by detection with a peroxidase-conjugated secondary antibody using chemiluminescence. Membranes that were stripped for loading controls were reprobed with goat polyclonal anti-β-actin antibody (1∶1000; Santa Cruz Biotechnology, Santa Cruz, CA). Proteins were quantified by using ImageGauge software version 4.22 (Fuji Film).

### MLV Infectivity Assay

As described above, virus particles were produced using transient transfection of 293T cells with viral expression plasmids. In experiments where the ability of HECT Ub ligases or Ub ligase fragments was used to enhance or inhibit viral release, various amounts of YFP-fusion protein expression plasmid were cotransfected with the viral constructs as indicated. At 24 h post-transfection the cell culture medium was clarified by low speed centrifugation and passed through a 0.45-µm-pore-size syringe filter. Subconfluent cultures of DFJ8 target cells were infected with dilutions of clarified media containing virus. At 24 h after infection, β-galactosidase activity in infected target cells was determined with a β-galactosidase assay kit (Stratagene) and amounts of infectious MLV produced were quantified by counting blue stained target cells.

## Results

### L-domain Activity of Core PPAY and PNAP Motifs in the Context of MLV

**Figure 1:**
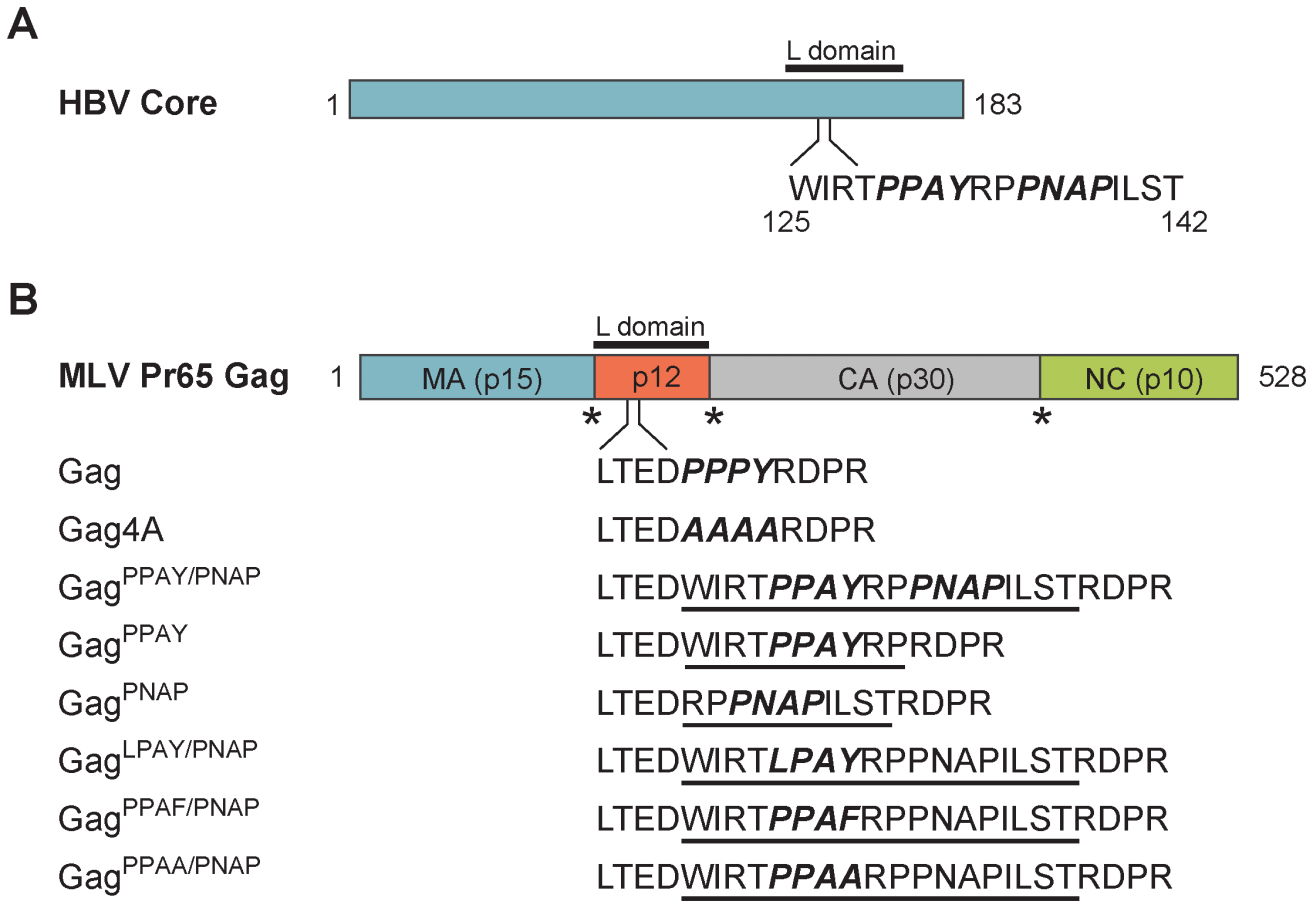
Generation of MLV Gag^Core^ L-domain chimeras. (A) HBV Core encodes a putative L-domain motif suggesting a potential docking site for HECT Ub ligases that direct HBV release. (B) L-domain chimeric proteins between MLV Gag and Core were generated by replacement of the endogenous MLV PPPY motif with the Core PPAY/PNAP motif and flanking residues. The location of the MLV Gag PPPY motif in the p12 region is italicized. The Gag4A mutant contains four alanine amino acid substitutions (AAAA) that abolish Gag L-domain activity. The inserted Core sequence is underlined in the MLV L-domain chimeras. The L-domain motif and L-domain motif mutations are italicized. Chimeric Gag protein was subcloned into the MLV GagPol expression plasmid to generate GagPol, GagPol4A, GagPol^PPAY/PNAP^, GagPol^PPAY^, GagPol^PNAP^, GagPol^LPAY/PNAP^, GagPol^PPAF/PNAP^, and GagPol^PPAA/PNAP^ expression constructs. The MLV Pr65 Gag protein contains matrix (MA), capsids (CA), and nucleocapsid (NC) domains. Asterisks indicate PR cleavage sites.

**Figure 2:**
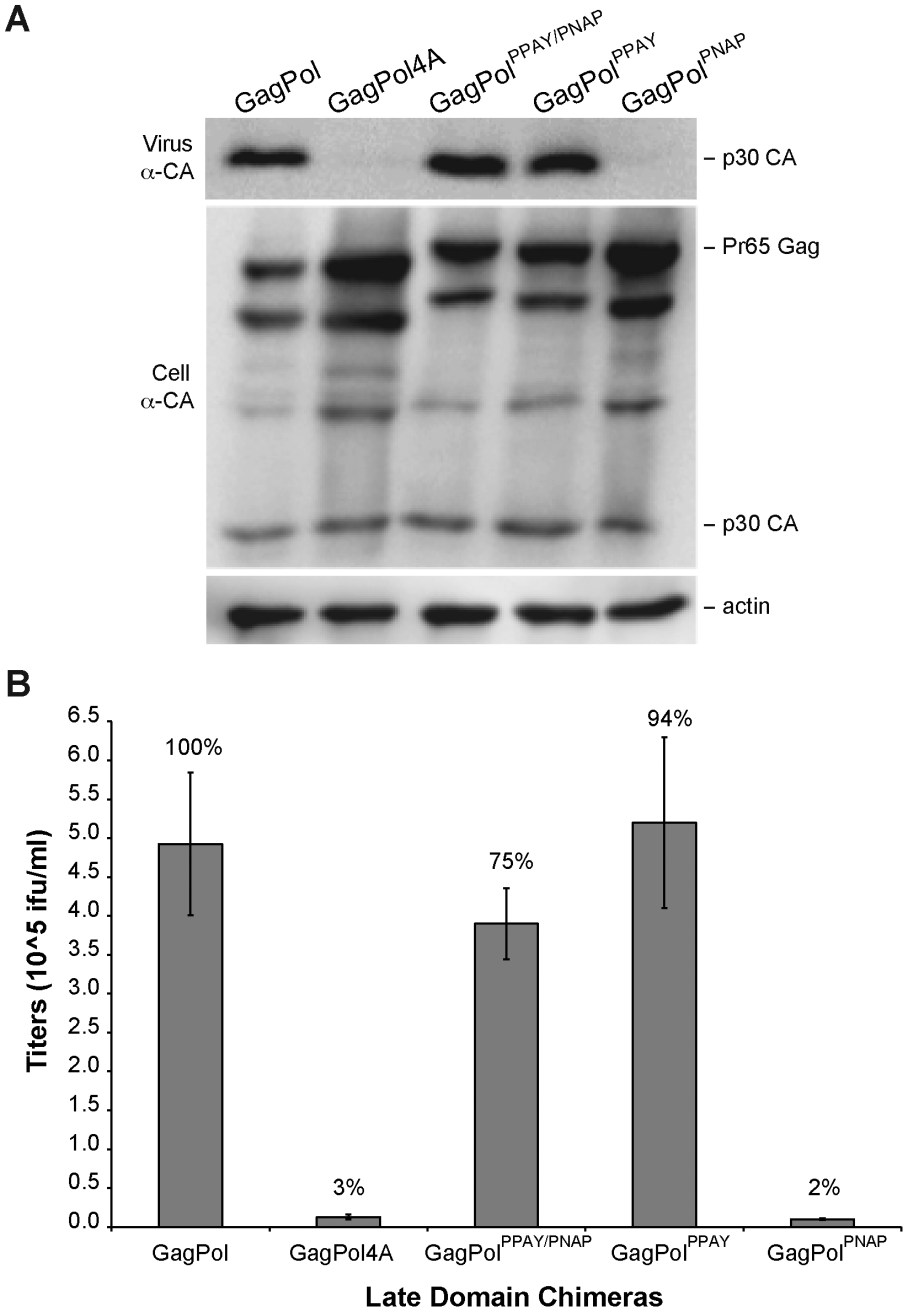
Core PPAY motif mediates release of infectious virus in the context of MLV Gag. (A) Virion production by 293T cells co-transfected with plasmids Friend MLV Env, MLV LTR-LacZ, and GagPol, GagPol4A, or chimeric GagPol containing the indicated L-domain motifs from Core. Released virus and cellular Gag protein levels were detected by western blot analysis with MLV Gag antibodies. Equal loading of cellular lysates was compared to β-actin protein expression. (B) Effect of indicated Core L-domain motif on infectious MLV production. Indicated percentages are relative to the infectious virus production observed in MLV with the endogenous PPPY motif. The mean and standard error of three independent experiments are indicated.

To determine if the Core _129_PPAYRPPNAP^138^ sequence constitutes a genuine L-domain motif that directs release of a heterologous virus, expression constructs were generated to encode an MLV GagPol^Core^ chimera protein where the endogenous PPPY L-domain of Gag was replaced with the putative Core L-domain motif (Fig. 1A and B). An MLV GagPol^PPAY/PNAP^ chimera was synthesized by insertion of a Core 18 amino acid sequence fragment containing both the PPAY and PNAP motifs, as well as two MLV GagPol constructs that contained a 10 amino acid sequence with the PPAY or the PNAP sequence motif, GagPol^PPAY^ and GagPol^PNAP^, respectively. To produce quantifiable infectious MLV particles containing chimeric protein, 293T cells were transfected with plasmids encoding the Friend MLV envelope protein, the LTR-LacZ reporter construct and the GagPol plasmids encoding either wild-type Gag, a Gag4A mutant that lacks L-domain activity, Gag^PPAY/PNAP^, Gag^PPAY^, or Gag^PNAP^. Media and cellular lysates were harvested from virus-replicating cells 1 day post-transfection and resolved by SDS-PAGE to detect virion-associated Gag or cell-associated Gag protein, respectively, by western blot (Fig. 2A). Additionally, DFJ8 target cells that stably express MCAT-1, a surface receptor that is recognized by the Friend MLV envelope protein, were incubated with serial dilutions of the collected media for 24 h and subsequently stained for LacZ activity to quantify the amount of infectious MLV particles produced by the virus-replicating cells (Fig. 2B). As previously reported [39], [42], the AAAA (4A) mutation in Gag strongly inhibited infectious MLV release to 3% of wild-type MLV (Fig. 2A and B). Interestingly, the 18 amino acid sequence of Core that contains the PPAY and PNAP motif restored release of infectious MLV to 75% of wild-type MLV. Functional replacement of the endogenous Gag PPPY motif by the Core sequence can be attributed to the PPAY motif since that sequence alone can induce MLV release at levels comparable to wild-type Gag while the PNAP sequence alone lowers MLV release to levels similar to that observed for the 4A mutation (Fig. 2A and B).

### Chimeric L-domain Mutants Reduce Production of Infectious Virions

**Figure 3:**
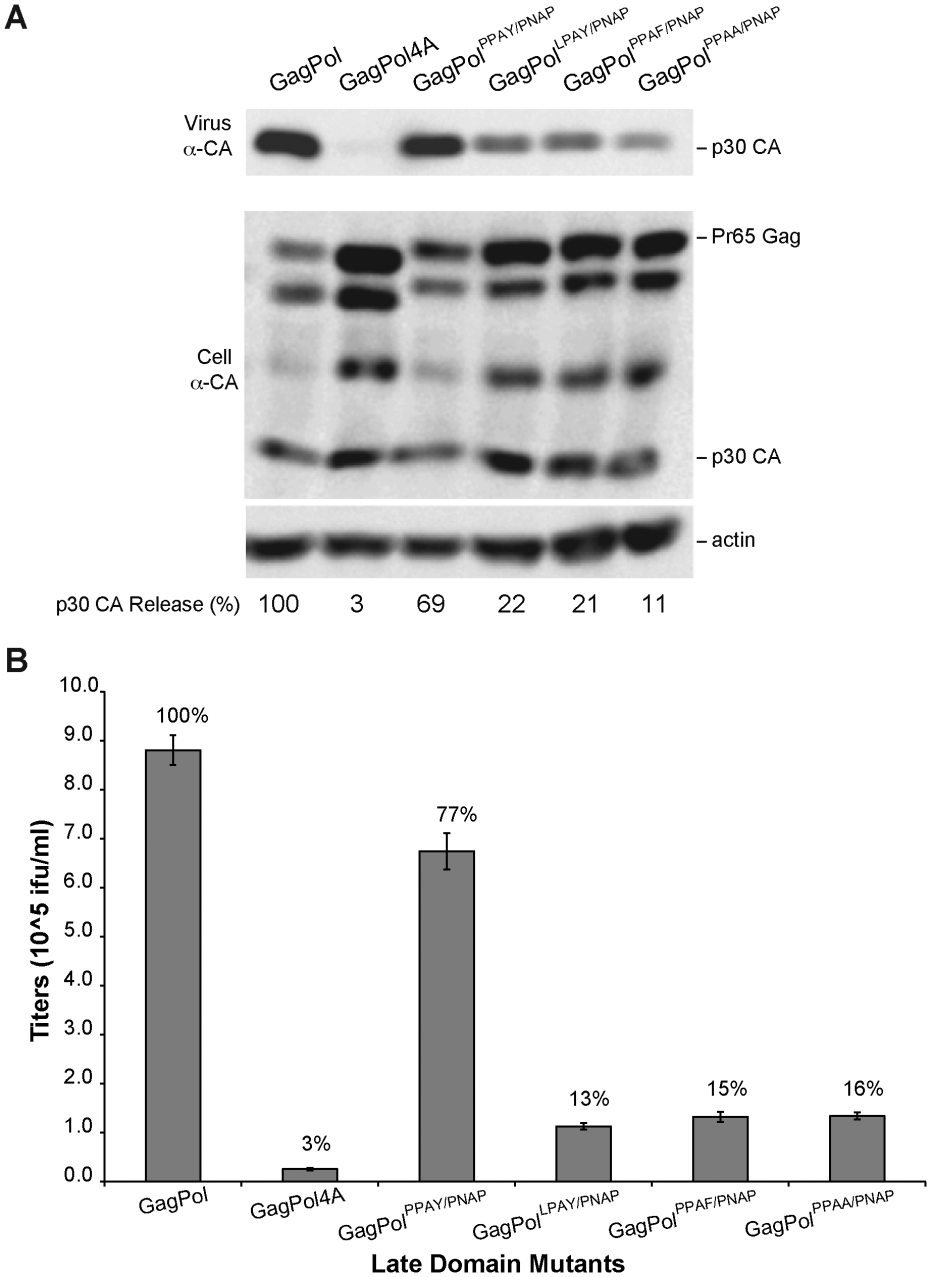
Core L-domain mutations reduce production of infectious MLV virions. (A) Virion production by 293T cells co-transfected with plasmids Friend MLV Env, MLV LTR-LacZ, and GagPol, GagPol4A, GagPol^PPAY/PNAP^, or the chimeric GagPol containing the indicated Core PPAY sequence mutations. Released virus and cellular Gag protein levels were detected by western blot analysis with MLV Gag antibodies. Equal loading of cellular lysates was compared to β-actin protein expression. (B) Effect of indicated Core PPAY sequence mutations on infectious MLV production. Indicated percentages are relative to the infectious virus production observed in MLV with the endogenous PPPY motif. The mean and standard error of three independent experiments are indicated.

To determine if the PPAY residues are critical for mediating the L-domain activity in the MLV Gag^Core^ chimera, single site mutations were generated in the MLV GagPol^PPAY/PNAP^ construct (Fig. 1B). Two constructs were generated that contained conservative mutations with leucine for proline at Core position 129 (GagPol^LPAY/PNAP^) and with tyrosine for phenylalanine at Core position 132 (GagPol^PPAF/PNAP^). An additional construct was made with tyrosine 132 mutated to alanine (GagPol^PPAA/PNAP^) as this mutation abolishes HBV capsid assembly and therefore cannot be analyzed in the context of replicating HBV [31], [34]. As described above, infectious MLV chimeric particles were produced in 293T cells by co-expressing plasmids encoding the Friend MLV envelope protein, the LTR-LacZ reporter construct and GagPol plasmids encoding wild type Gag, Gag4A, Gag^PPAY/PNAP^, Gag^LPAY/PNAP^, Gag^PPAF/PNAP^, or Gag^PPAA/PNAP^. Media and cellular lysates were harvested from virus-replicating cells 1 day post transfection and resolved by SDS-PAGE to detect virion-associated Gag or cellular-associated Gag protein by western blot (Fig. 3A). Harvested media was also used to infect target cells before staining for LacZ activity to quantitate the release of infectious MLV particles from virus-replicating cells (Fig. 3B). As predicted, mutations within the PPAY motif inhibited release of infectious virions as observed by reduced staining of target cells (Fig. 3B). The chimeric L-domain mutants exhibited a partial release phenotype with infectious virus release at 15% of wild-type MLV as compared to the GagPol4A mutant, which was at 3% of wild-type MLV. This suggests that the chimeric L-domain mutants contain residual binding activity for host proteins that allow low-level viral release, and supports the authenticity of the Core L-domain motif.

### Nedd4 or WWP1 Rescues the Release of Chimera Mutants in an L-domain-dependent Manner

Partial release defects of MLV L-domain mutants can be rescued by overexpression of Nedd4 Ub ligase family members [19]. To identify HECT ligases that may be recruited by the L-domain in MLV GagPol^Core^ chimeras, a functional screen was conducted by titrating in increasing concentrations (ranging from 100 ng to 2000 ng) of full-length expression constructs for the Nedd4 Ub ligase family members Nedd4, Nedd4L, WWP1, WWP2, Smurf1, Smurf2, and Itch in cells co-expressing wild-type GagPol, GagPol4A, GagPol^PPAY/PNAP^, or the GagPol^PPAF/PNAP^ partial release mutant. The effects on infectious virion release and virion-associated Gag proteins were then examined. Three criteria were applied to identify the specific HECT ligases that are important for L-domain dependent release of the MLV GagPol^PPAF/PNAP^ mutant chimera (Table 1). First, HECT ligase expression should increase the production of infectious MLV containing the partial release PPAF/PNAP domain. Second, the increase in infectious virus release should correspond to an increase in the amount of the mature p30 CA Gag proteolytic product. Third, release due to HECT ligase overexpression should be exclusively L-domain dependent and thus not have an effect on the release of the GagPol4A mutant that lacks a functional L-domain.

**Table 1:** Summary of functional screen with full-length HECT ligases.

HECT Ligase	Increased infectivity	Increased release of GagPol^PPAF/PNAP^ p30	Increased release of GagPol4A p30
Nedd4^1^	Yes	Yes	No
Nedd4L	Yes	Yes	Yes
WWP1^1^	Yes	Yes	No
WWP2	No	No	No
Smurf1	No	Yes	No
Smurf2	Yes	No	No
Itch	Yes	Yes	Yes

1 HECT ligases that meet all three screening criteria.

**Figure 4:**
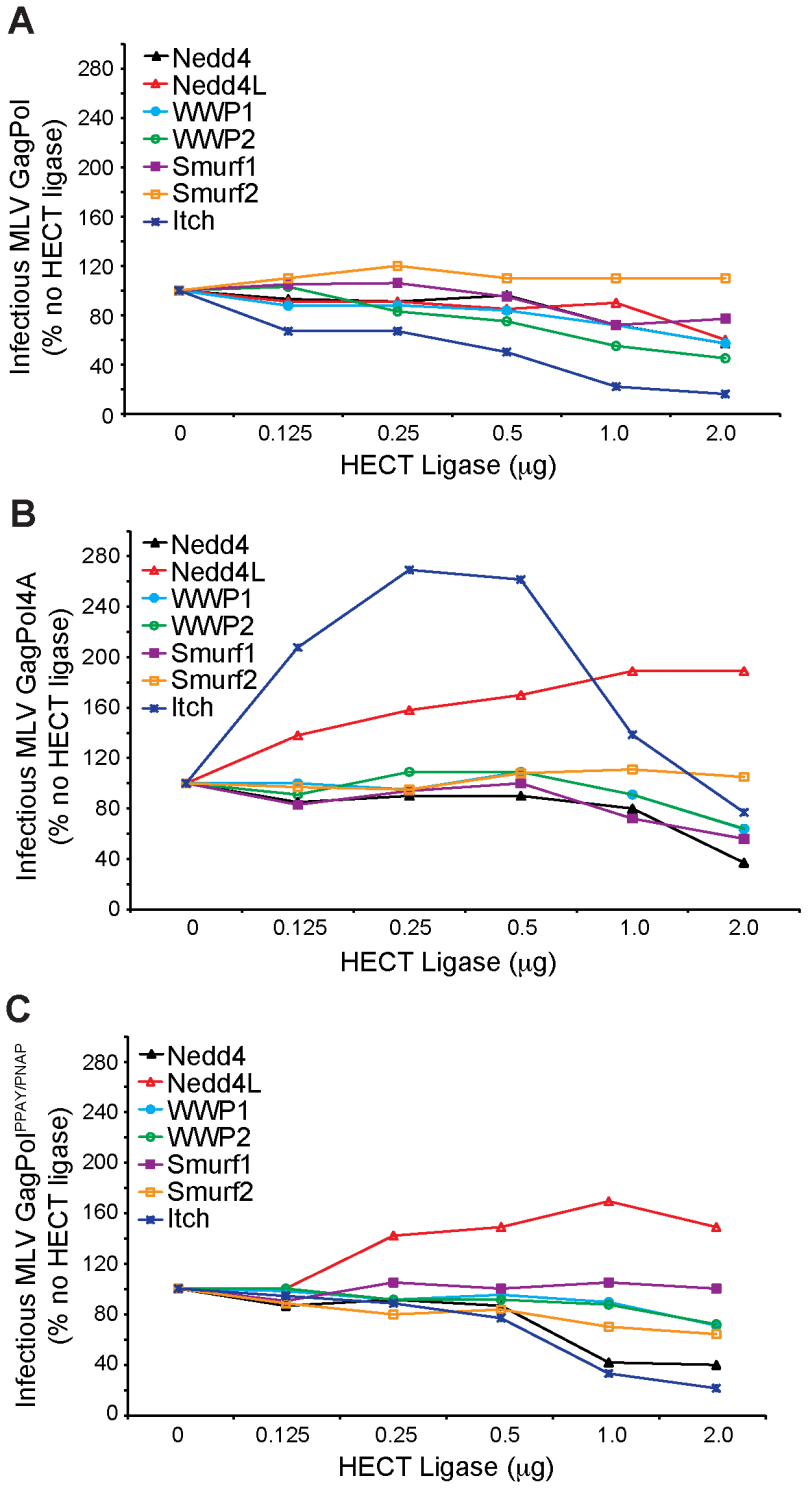
Effect of HECT ligase overexpression on infectious MLV production. Virion production by 293T cells co-transfected with plasmids Friend MLV Env, MLV LTR-LacZ, and (A) GagPol, (B) GagPol4A, (C) GagPol^PPAY/PNAP^ alone or with increasing concentrations (0.125 µg to 2 µg) of a YFP fusion expression construct encoding Nedd4, Nedd4L, WWP1, WWP2, Smurf1, Smurf2 or Itch. Percentages of infectious virus are relative to infectious virus produced when not co-expressed with the indicated HECT ligase.

Analysis of the effect of HECT ligase overexpression on infectious MLV release demonstrated that the seven HECT ligases tested did not enhance, and at high concentrations at which cytotoxic effects are expected, reduced infectious MLV production in wild-type MLV GagPol expressing cells (Fig. 4A). This result is to be expected as wild-type MLV GagPol contains a functional L-domain and is efficiently released at normal cellular levels of HECT ligase expression. In GagPol4A expressing cells, both Nedd4L and Itch overexpression enhanced the production of infectious virus indicating that they are functioning in an L-domain independent manner (Fig. 4B), similar to previous reports [43]. In the context of cells expressing the GagPol^PPAY/PNAP^ L-domain chimera, six of the seven HECT ligases screened had no effect on the production of infectious MLV (Fig. 4C), similar to MLV GagPol. The exception was Nedd4L, which similar to the effect observed in cells expressing GagPol4A enhanced infectious MLV production, presumably in an L-domain independent manner.

**Figure 5:**
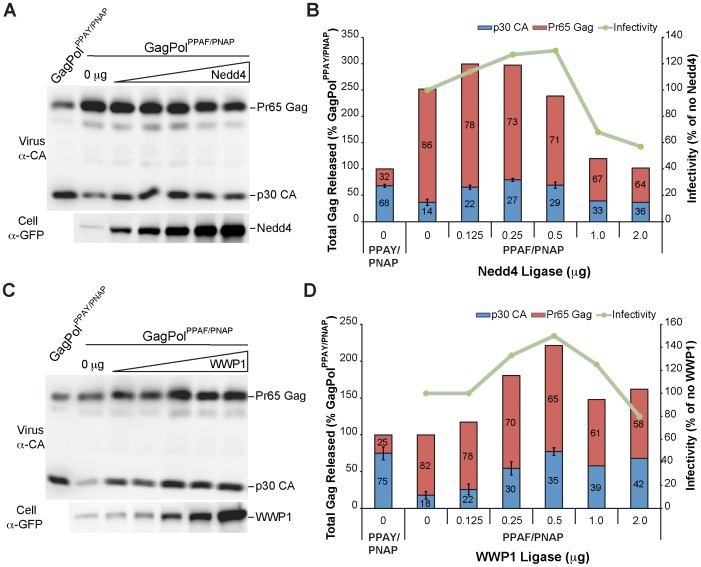
Nedd4 and WWP1 overexpression rescues release of an MLV L-domain chimera mutant. Virion production by 293T cells co-transfected with plasmids Friend MLV Env, MLV LTR-LacZ, and GagPol^PPAY/PNAP^ alone, GagPol^PPAF/PNAP^ alone, or GagPol^PPAF/PNAP^ with increasing concentrations (0.125 µg to 2 µg) of a YFP fusion expression construct encoding (A and B) Nedd4 or (C and D) WWP1. Released virus and HECT ligase protein levels were detected by western blot analysis with the indicated antibodies. (B and D) Protein levels for Pr65 Gag (immature; red bar) and p30 CA (mature; blue bar) were quantified as described above. The mean and standard error of two independent experiments are indicated. Infectious MLV virus produced from the harvested samples was measured and compared to the production of Gag protein products in the released virus samples. Percentages of infectious virus are relative to infectious virus produced without coexpression of the indicated HECT ligase.

The HECT ligases were next assayed for the ability to stimulate release of infectious virions and the mature p30 CA Gag product using the GagPol^PPAF/PNAP^ partial release mutant. In the absence of HECT ligase overexpression, MLV GagPol^PPAF/PNAP^ demonstrated a >80% reduction of released mature virions as indicated by p30 CA protein levels when compared to MLV GagPol^PPAY/PNAP^ (Fig. 3). When Nedd4 (Fig. 5A and B) or WWP1 (Fig. 5C and D) were titrated in at increasing concentrations, the total level of Gag released by MLV GagPol^PPAF/PNAP^ into the media maximally increased compared to MLV GagPol^PPAF/PNAP^ in the absence of ligase (Fig. 5B and D). The total level of released Gag eventually decreased at higher concentrations of overexpressed HECT ligase most likely due to disruption of the endocytic trafficking pathways that are regulated by HECT ligase activity. Additionally, the level of released mature virions for MLV GagPol^PPAF/PNAP^ increased to levels comparable to the MLV GagPol^PPAY/PNAP^ chimera with co-expression of 500 ng of the indicated HECT ligase. The release of mature virions correlated with infectious MLV production, indicating that Nedd4 and WWP1 overexpression can functionally rescue the release defect in an MLV L-domain chimera mutant.

**Figure 6:**
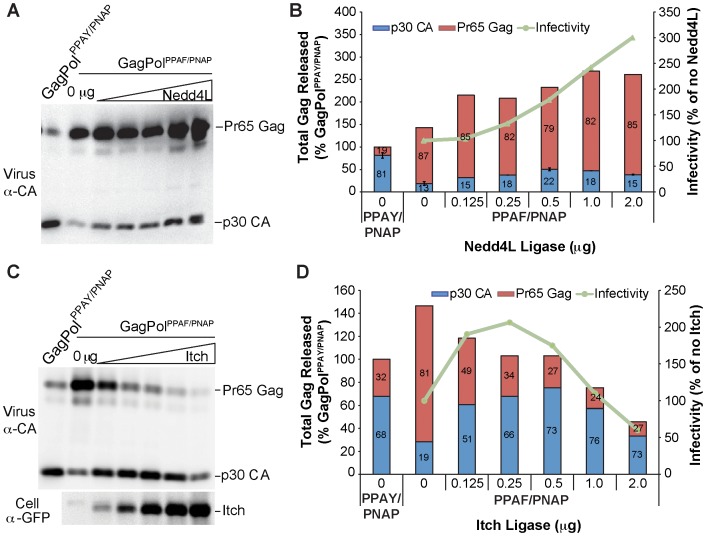
Nedd4L rescues release of an MLV L-domain chimera mutant and Itch corrects processing defects of MLV L-domain chimera mutant to enhance release of infectious virions. Virion production by 293T cells co-transfected with plasmids Friend MLV Env, MLV LTR-LacZ, and GagPol^PPAY/PNAP^ alone, GagPol^PPAF/PNAP^ alone, or GagPol^PPAF/PNAP^ with increasing concentrations (0.125 µg to 2 µg) of a YFP fusion expression construct encoding (A and B) Nedd4L or (C and D) Itch. Released virion-associated Gag proteins were detected with the indicated antibodies. (B and D) Protein levels for Pr65 Gag (immature; red bar) and p30 CA (mature; blue bar) were quantified as described above. Infectious MLV virus produced from the harvested samples was measured and compared to the production of Gag protein products in the released virus samples as described above.

Interestingly, Nedd4L also enhanced infectious MLV production by GagPol^PPAF/PNAP^ expressing cells that correlated with an increase in virion-associated p30 CA levels (Fig. 6A and B). Similar to what was observed for the GagPol4A mutant, overexpression of Itch in GagPol^PPAF/PNAP^ expressing cells significantly enhanced production of infectious MLV that correlated with an increase of p30 CA levels (Fig. 6C and D). Unlike WWP1, Nedd4, and Nedd4L however, increased p30 CA was associated with decreased Pr65 Gag, indicative of enhanced processing of the GagPol^PPAF/PNAP^ polyprotein precursor (Fig. 6C). Nevertheless, the observation that Nedd4L and Itch also enhance infectivity of the GagPol4A mutant (Fig. 4B) indicates they act in an L-domain-independent manner. In contrast to Nedd4, WWP1, Nedd4L, and Itch, overexpression of WWP2, Smurf1, or Smurf2 did not result in a consistent increase in virion-associated p30 CA levels and/or infectious MLV production (data not shown). Overall, the observation that Nedd4 and WWP1 specifically enhance infectious MLV production in GagPol^PPAF/PNAP^ but not GagPol4A expressing cells, unlike Nedd4L and Itch, indicates they are potential binding partners for the MLV L-domain chimera.

### MLV L-domain Chimera Release is Inhibited by WWP1 and Itch Fragments

**Figure 7:**
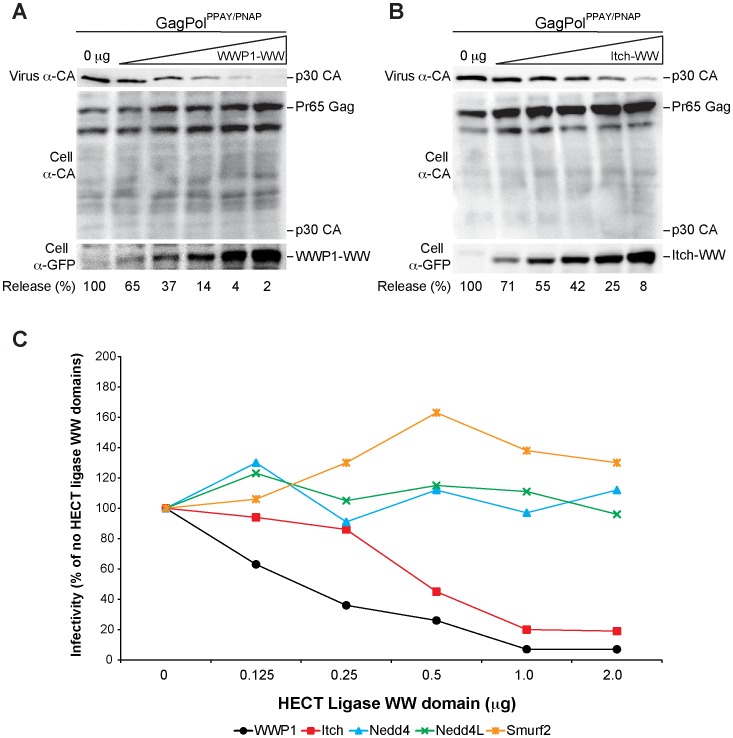
Inhibition of MLV L-domain chimera release by WWP1 and Itch fragments. Virion production by 293T cells co-transfected with plasmids Friend MLV Env, MLV LTR-LacZ, and GagPol^PPAY/PNAP^ alone or GagPol^PPAY/PNAP^ with increasing concentrations (0.125 µg to 2 µg) of the indicated expression plasmids encoding the YFP-WW domain fragment of (A) WWP1 or (B) Itch. Released virus and cellular protein levels were detected by western blot analysis with the indicated antibodies. Percentage of released virus is relative to virus released without coexpression of the indicated WW domain fragment. (C) Effect of YFP-WW domain fragments from WWP1, Itch, Nedd4, Nedd4L, and Smurf2 on infectious GagPol^PPAY/PNAP^ production when co-expressed at increasing amounts. Percentages of infectious GagPol^PPAY/PNAP^ particles are relative to infectious GagPol^PPAY/PNAP^ particles produced without coexpression of the indicated WW domain fragment.

The ability of Nedd4 and WWP1 to restore release of a partial release L-domain chimera mutant indicates these proteins may bind to the Core PPAY domain in the MLV L-domain chimera to mediate viral release. To determine if Nedd4 and WWP1 restored release by specifically binding to the L-domain mutant motif, the inhibitory effect of dominant negative WW domain fragments from HECT ligases were tested in cells replicating the MLV GagPol^PPAY/PNAP^ chimera. Expression plasmids encoding YFP-WW-domain fusion proteins from WWP1, Itch, Nedd4, Nedd4L, or Smurf2 were titrated in cells co-expressing MLV GagPol^PPAY/PNAP^ and the production of infectious virus released in the media was assayed (Fig. 7). Of the five constructs assayed, only the YFP-WW-domain fusion proteins based on Itch and WWP1 significantly inhibited infectious virion production (Fig. 7).

## Discussion

These studies show that an HBV Core amino acid sequence containing the PPAY motif can functionally substitute for the MLV Gag L-domain to promote viral release by recruitment of Nedd4 family Ub ligases. Given that some L-domains from different viruses are functionally interchangeable, the use of L-domain swapping experiments can serve as a tool to identify viral sequences that exhibit L-domain activity [35]. By using the retrovirus MLV Gag structural protein to test an 18 amino acid proline-rich sequence (_125_WIRTPPAYRPPNAPILST^142^) of HBV Core, we found that the PPAY motif at positions 129–132 was able to promote viral release and thus exhibits L-domain activity. Mutation of key motif residues within the L-domain chimeras (GagPol^LPAY/PNAP^, GagPol^PPAF/PNAP^, and GagPol^PPAA/PNAP^) reduced viral release, further supporting the argument for L-domain activity by the HBV Core sequence. While it is intriguing that an additional L-domain-like motif is found adjacent to the PPAY motif, a PNAP sequence at position 135–138, this sequence did not exhibit L-domain activity. Although not the canonical P(T/S)AP sequence, the PNAP motif has previously been described in 5-lipoxygenase as a potential interaction site for cytoskeletal proteins and proline-rich SH3 binding domain in Grb2, an adaptor protein for tyrosine kinase-mediated cell signaling [44]. The observation that some L-domains are context-dependent suggests that the PNAP sequence may demonstrate L-domain activity in PT(S)AP-containing viruses, such as HIV-1, that interact with the SH3 domain of Tsg101 for release [28]. However, there are no previous reports of viral L-domains containing a PNAP motif and a sequence alignment study with over 400 Core protein sequences across HBV genotypes has revealed that the P135 residue is not conserved [45]. In contrast, the P129 and Y132 residues in the PPAY motif are highly conserved suggesting the PPAY motif, and not the PNAP motif, is important for mediating HBV release.

The levels of virus release by the GagPol^LPAY/PNAP^, GagPol^PPAF/PNAP^, and GagPol^PPAA/PNAP^ L-domain mutant chimeras, while less than wild-type MLV, was not as strongly reduced as that observed for the GagPol4A mutant that lacks L-domain activity. This indicates that the L-domain mutants demonstrate a partial release phenotype that may be attributed to residual L-domain binding of host factors that direct viral release. Such L-domain mutants have been previously used to identify HECT ligases that can restore release and are therefore identified as potential binding partners for the viral L-domain motif [19]. After employing a similar functional screen with seven HECT ligases from the Nedd4 Ub ligase family, we found that overexpression of Nedd4 and WWP1 restored release of GagPol^PPAF/PNAP^ as demonstrated by the increased levels of virion-associated p30 CA, which is indicative of mature virion release. Interestingly, while the total level of virion-associated Gag increased with Nedd4 and WWP1 overexpression, the ratio of p30 CA to Pr65 Gag also increased with a correlative increase in the infectivity of target cells. Although maximum target cell infectivity by virions produced in cells expressing the L-domain chimera mutant corresponded to the peak production of virion-associated p30 CA, infectivity did not reach levels comparable to the GagPol^PPAY/PNAP^ chimera that contains a functional L-domain (data not shown). This was unexpected considering that maximum p30 CA levels with Nedd4 or WWP1 ligase overexpression were comparable to the p30 CA levels released by GagPol^PPAY/PNAP^.

We attempted to confirm that Nedd4 and WWP1 enhanced release of GagPol^PPAF/PNAP^ in a PPxY-dependent manner by overexpressing ligase WW-domain fragments in GagPol^PPAY/PNAP^ to determine if release would be inhibited. Surprisingly, the Nedd4 WW-domain did not inhibit release of the L-domain chimera. While it may be that Nedd4 binds to the Core L-domain but is not required for promoting viral release similar to what is observed in HIV-1 and Ebola L-domain chimera studies [28], the existence of other L domains within MLV may also be masking the effects of the Nedd4 dominant negative expression. Indeed, Nedd4 promotes viral release in an L-domain dependent manner in wild-type MLV, but overexpression of dominant negative constructs of Nedd4 results in negligible reductions in viral release, an effect that can be overcome by mutating Gag sequences required for the binding of L-domain interacting proteins Tsg101 and Alix [30]. As expected, the WW-domain of the WWP1 ligase inhibited release of the L-domain chimera, which supports WWP1 as a potential binding partner for the HBV Core PPAY motif. However, because of the potent effects of WWP1 dominant negative expression in wild-type MLV [19], further investigation into the effects of WWP1 during HBV replication are needed to determine if it truly interacts with the HBV Core PPAY motif to promote HBV release.

In contrast to Nedd4 and WWP1 overexpression, Itch overexpression did not increase the total level of virion-associated Gag produced by GagPol^PPAF/PNAP^ but instead was comparable to the total level of Gag produced by GagPol^PPAY/PNAP^. Additionally, low levels of Itch overexpression significantly reduced the amount of Pr65 Gag released by GagPol^PPAF/PNAP^ and significantly increased p30 CA levels. The maximum level of p30 CA produced by GagPol^PPAF/PNAP^ due to Itch overexpression was comparable to p30 CA levels released by the functional L-domain chimera, but unlike Nedd4 and WWP1 overexpression, Itch enhanced virion infectivity to significantly higher levels and decreased virion infectivity of wild-type MLV and the GagPol^PPAY/PNAP^ L-domain chimera. Surprisingly, Itch overexpression also significantly enhanced production of infectious virions released by GagPol4A expressing cells indicating this effect could act in an L-domain independent manner. The observation that low levels of Itch overexpression significantly increases infectivity for both GagPol^PPAF/PNAP^ and GagPol4A suggests that Itch may correct Gag processing defects of the MLV L-domain mutants to enhance release of infectious virions. However, in contrast to the WW-domain of Nedd4L ligase that also acts in an L-domain independent manner to stimulate release of GagPol4A, overexpression of the Itch WW-domain in GagPol^PPAY/PNAP^ replicating cells significantly inhibited release of the functional L-domain chimera demonstrating that Itch can act in an L-domain dependent manner as well. These results suggest that Itch can rescue the release defects of L-domain mutants by associating with Gag either directly through the PPxY motif or through another as yet unidentified motif within Gag, and/or indirectly through Gag-associated proteins.

Despite recent evidence supporting the involvement of the cellular ubiquitin pathway, the exact mechanisms and host factors involved in HBV assembly and release are still not fully understood. Microscopy studies have demonstrated that HBV proteins traffic to intracellular vesicles reminiscent of MVBs or late endosomes [46], [47]. During normal cellular processes, MVBs are involved in regulating cell signaling and receptor recycling by sorting and trafficking endocytosed bodies to the lysosome for degradation or outside the cell as exosomes. Formation of intralumenal vesicles within MVBs involves the transient recruitment of the ESCRT machinery to the MVB membrane (reviewed in [48]). Disassociation of the ESCRT complex occurs through the actions of additional proteins including ATPase Vps4 [48], [49]. Studies have shown that HBV proteins can associate with Nedd4 and/or γ2-adaptin, cellular proteins that can interact with components of the ESCRT machinery [31], [50], [51]. Additionally, HBV maturation is sensitive to inhibition of ESCRT-II, ESCRT-III and Vps4 [52]–[55], further supporting a model of HBV release that involves at least portions of the MVB pathway.

Although an interaction between Nedd4 and HBV Core had previously been demonstrated [31], the role of Nedd4 and its related ligases in HBV replication is not completely clear. In other PPAY motif-containing viruses, Nedd4 Ub ligases can promote access to the endocytic machinery required for viral release through their interaction with the viral L domains [21]. Our confirmation that the HBV core PPAY motif exhibits genuine L domain activity mediated by interactions with Nedd4 Ub ligases provides further evidence that Nedd4 and/or WWP1 may play a similar role during HBV replication. However, the exact mechanisms by which Nedd4 Ub ligases may promote HBV viral egress remains to be elucidated. The absence of direct ubiquitilation of Core [31], [56] suggests a model by which Nedd4 family ligases serve as intermediates to interact with components of the ESCRT machinery either directly or through development of a Nedd4-γ2-adaptin complex as proposed in [55]. Additionally, it remains unclear how Nedd4 ubiquitin ligases would gain access to the PPAY motif because this region of Core is essential for the formation of interdimer contacts and would normally be concealed within the viral particle [32], [33]. Stability and/or conformational changes of the viral capsid that occur after or during retrotranscription of the viral genome [32], [57] may permit host proteins at least temporary access to the PPAY motif. Furthermore, the required duration and stoichiometry for the L-domain-HECT ligase interaction to have physiological consequences is unknown, and minimal contact may suffice for proper regulation of viral release.

Retrovirus assembly and release is traditionally thought to occur primarily at the plasma membrane and is therefore a distinct pathway from the intracellular assembly and envelopment of HBV virions. However, recent studies have shown that retroviral release can also occur through the MVB pathway [30], [39], [58]. Although the studies discussed here were conducted in a retrovirus system, they serve to confirm the authenticity of the Core PPAY motif as a functional L-domain and to identify Nedd4 ubiquitin ligases that serve as potential binding partners for the Core PPAY motif that might promote HBV release. Further studies in an HBV system may reveal that pharmacological inhibition of cellular proteins that direct HBV release such as Nedd4 and WWP1 represents a new therapeutic approach for blocking virus replication and treating chronic HBV infection.
